# Exploring and Identifying Prognostic Phenotypes of Patients with Heart Failure Guided by Explainable Machine Learning

**DOI:** 10.3390/life12060776

**Published:** 2022-05-24

**Authors:** Xue Zhou, Keijiro Nakamura, Naohiko Sahara, Masako Asami, Yasutake Toyoda, Yoshinari Enomoto, Hidehiko Hara, Mahito Noro, Kaoru Sugi, Masao Moroi, Masato Nakamura, Ming Huang, Xin Zhu

**Affiliations:** 1 Biomedical Information Engineering Lab, The University of Aizu, Aizuwakamatsu 965-8580, Japan; d8212108@u-aizu.ac.jp; 2 Division of Cardiovascular Medicine, Toho University Ohashi Medical Center, Tokyo 153-8515, Japan; naohiko.sahara@med.toho-u.ac.jp (N.S.); masako.asami@med.toho-u.ac.jp (M.A.); yasutake.toyoda@med.toho-u.ac.jp (Y.T.); yonomo1225@oha.toho-u.ac.jp (Y.E.); harahide@oha.toho-u.ac.jp (H.H.); moroi@med.toho-u.ac.jp (M.M.); masato@oha.toho-u.ac.jp (M.N.); 3 Division of Cardiovascular Medicine, Odawara Cardiovascular Hospital, Odawara 250-0873, Japan; noro@oha.toho-u.ac.jp (M.N.); sugi@ojh.or.jp (K.S.); 4 Division of Information Science, Nara Institute of Science and Technology, Ikoma 630-0192, Japan; alexmhuang@is.naist.jp

**Keywords:** heart failure, machine learning, mortality risk, patient phenotypes, prognosis

## Abstract

Identifying patient prognostic phenotypes facilitates precision medicine. This study aimed to explore phenotypes of patients with heart failure (HF) corresponding to prognostic condition (risk of mortality) and identify the phenotype of new patients by machine learning (ML). A unsupervised ML was applied to explore phenotypes of patients in a derivation dataset (n = 562) based on their medical records. Thereafter, supervised ML models were trained on the derivation dataset to classify these identified phenotypes. Then, the trained classifiers were further validated on an independent validation dataset (n = 168). Finally, Shapley additive explanations were used to interpret decision making of phenotype classification. Three patient phenotypes corresponding to stratified mortality risk (high, low, and intermediate) were identified. Kaplan–Meier survival curves among the three phenotypes had significant difference (pairwise comparison *p* < 0.05). Hazard ratio of all-cause mortality between patients in phenotype 1 (n = 91; high risk) and phenotype 3 (n = 329; intermediate risk) was 2.08 (95%CI 1.29–3.37, *p* = 0.003), and 0.26 (95%CI 0.11–0.61, *p* = 0.002) between phenotype 2 (n = 142; low risk) and phenotype 3. For phenotypes classification by random forest, AUCs of phenotypes 1, 2, and 3 were 0.736 ± 0.038, 0.815 ± 0.035, and 0.721 ± 0.03, respectively, slightly better than the decision tree. Then, the classifier effectively identified the phenotypes for new patients in the validation dataset with significant difference on survival curves and hazard ratios. Finally, age and creatinine clearance rate were identified as the top two most important predictors. ML could effectively identify patient prognostic phenotypes, facilitating reasonable management and treatment considering prognostic condition.

## 1. Introduction

Heart failure (HF) has a high prevalence worldwide and is estimated to affect 64.3 million people [1]. In developed countries, about 1–2% of adults suffer from HF [2] with the incidence of HF rapidly increasing with age [3]. Globally, Japan has the most rapidly aging society. In 2021, 29.4% of the population was aged 65 years or older [4,5], and the number of HF subjects is expected to reach 1.3 million by 2030 [6]. Furthermore, HF is a leading cause of hospitalization in Japan and imposes a heavy burden on society [7]. Although the prognosis for survival in Japanese patients with HF is better than that in European and US populations, the length of stay in Japan is approximately three times that of Western countries. A reduction in length of stay is necessary to alleviate the demand on the Japanese health care system caused by the HF pandemic [4]. Identifying patient phenotypes corresponding to prognosis, and making informed decisions including treatment, medication, length of stay, and even cost are very important [8].

Although some models, such as the Seattle heart failure model [9], have been developed for survival prediction, their generalization ability may be limited due to differences in treatments among populations in different regions and countries [10]. For example, some treatments, both pharmacological and nonpharmacological, are unique to Japan [4]. Therefore, Miyagawa et al. developed and validated a Japan heart failure model for the 5-year survival prediction of Japanese patients with HF [10]. This model mainly considers patients with an ejection fraction less than 35% and does not consider the phenotypes with stratified mortality risk nor the specific significant factors associated with mortality for phenotypes in different risk levels. Additionally, exploring and identifying patient phenotypes by manually reviewing medical records is difficult. Machine learning (ML) techniques have been applied for varied HF-related issues or tasks in prior studies, such as detection, classification, and prediction of medication adherence [11,12]. In addition, ML methods had considerable ability to predict short-term survival of patient with HF using serum creatinine and ejection fraction only [13]. Notably, an unsupervised ML method has performed considerable potential to identify patient phenotypes. Segar et al. used a penalized finite-mixture-model-based clustering analysis to successfully identify three phenotypes with distinct clinical characteristics and long-term outcomes of patients with HF with preserved ejection fraction [14]. Bretos-Azcona et al. demonstrated the existence of distinct three risk subgroups within population of high-risk multiple chronic condition patients using a clustering method, and suggested subgroup-specific treatment strategy instead of a uniform one [15]. In addition, Stevens et al. declared unsupervised ML could provide a basis for homogenization and was expected to guide personalized intervention [16]. More recently, Inomata et al. developed a smartphone-based phenotyping method using ML clustering to identify distinct subgroups with heterogeneous dry eye symptoms, and concluded that such a phenotyping method could guild precise medicine [17].

It was noteworthy that unsupervised ML was a powerful tool for exploring patient phenotypes, but it had limitations when applied for new patients compared with supervised methods. The purpose of this study was to explore phenotypes of patients with HF corresponding to prognostic condition (risk of mortality) and identify the phenotype for new patients combining unsupervised and supervised ML methods. Simultaneously, we explain the decision-making process of a supervised ML model and identify important predictors which contribute to discriminate phenotypes and their effects on model output. Furthermore, phenotype-specific clinical characteristics of patients and significant risk and protective factors associated with mortality were discussed.

## 2. Materials and Methods

### 2.1. Data Collection

A retrospective study was conducted to investigate risk stratification in hospitalized patients with HF diagnosed using the Japanese Healthcare Data System at Toho University Ohashi Medical Center between 7 April 2016 and 17 March 2019, constituting a derivation dataset. All patients were enrolled in this study. If a patient had several admissions, the data from the first hospitalization only were analyzed. Data from other admissions were excluded.

All patients generated diagnosis procedure combination (DPC) data, which were developed for the Japanese insurance reimbursement system. DPC data consist of admission and discharge summaries, and also include the following patient details: age, sex, body mass index (BMI), medical procedures, medical cost, daily records of drug administration, and activities of daily living (ADL), according to the components of the Barthel index at hospital admission and discharge. The patient needed complete assistance for available items was defined as low ADL. Patients’ characteristics, laboratory data, and echocardiographic data from electronic medical records were analyzed as well. Finally, after verifying consistency between DPC data and medical data, the database for this study was created. Details of collected patient information are summarized in Table S1. Then, an independent validation dataset was organized which consisted of data from patients admitted between 20 March 2019 and 16 March 2020 at Toho University Ohashi Medical Center.

The protocol for the study was prepared in accordance with the Declaration of Helsinki, and this study was approved by the Institutional Review Board and Ethics Committee of Ohashi Hospital, School of Medicine, Toho University (No. H19031).

### 2.2. Preprocessing and Statistical Analysis

The preprocessing and statistical analysis were similar to a previous work [18]. Firstly, the percentage of missing variables was checked. Variables which missed in more than 20% of patients were deleted [19]. Because multiple imputation is hard to convergent with relatively small sample size but high dimension of variables, a commonly used simple imputation was conducted in this study. Missing values of continuous and categorical variables were imputed with mean and mode, respectively. Available data were presented as mean ± standard deviation or frequency (percentage) depending on the type of variables. A two-tailed, unpaired Student’s t-test was used to assess the difference of continuous variables with a normal distribution. A Mann–Whitney test was employed for skewed continuous variables. Normality was tested using the Shapiro–Wilk test. Categorical data were compared using Pearson’s chi-square test or Fisher’s exact test, as appropriate. In statistical analysis, *p* < 0.05 was considered statistically significant. Data preprocessing was performed using Python (Python Software Foundation, Beaverton, OR, USA; version 3.7.7), and statistical analysis was conducted using R (R Foundation for Statistical Computing, Vienna, Austria; version x64 3.6.0).

### 2.3. Phenotype Exploration and Classification

Agglomerative hierarchical clustering [20] was applied to group patients in clusters based on their similarity of clinical presentation. Ward’s method was used as a linkage criterion, which minimized the sum of squared differences within all clusters [21]. The optimal number of clusters was determined based on dendrogram [15], scree plot [22], and elbow methods [23]. Dimension reduction was performed using uniform manifold approximation and projection [24]. To further explore clinical implications, three patient phenotypes were further identified depending on the risk of mortality in the above clusters [25]. The stability and robustness of the clustering results were assessed as previously described [15]. For stability assessment, all the patients were divided into five subgroups, similar to fivefold cross validation, and then the cluster algorithm was implemented on each subgroup. For evaluation of robustness, a K-means cluster model was developed on all the patients. The number of clusters in K-means was set to equal the optimal number of clusters in agglomerative hierarchical clustering to compare the percentage of patients in each cluster group. Significant differences were checked by the chi-square test.

**Figure 1:**
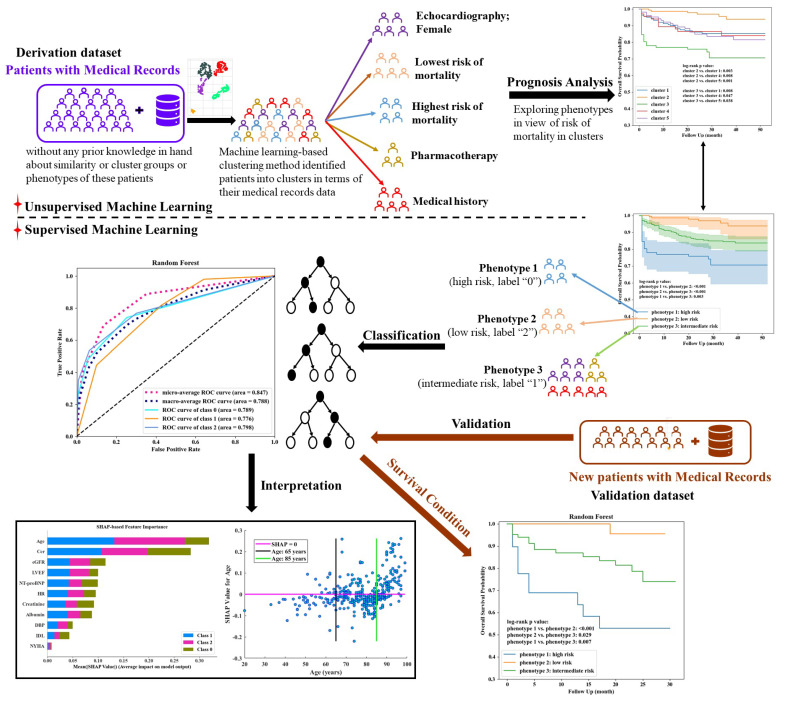
The proposed system frame for phenotype exploration and classification combining unsupervised and supervised machine learning methods.

The unsupervised clustering along with supervised ML method would have better reproducibility for new unknown patients [26]. Popular supervised learning methods such as decision tree and random forest were employed for phenotypes classification. In this way, the system could be directly applied to identify the phenotype of new patients. Variables performed significant difference among the three phenotypes were used as predictors for phenotypes classification. For model development, data in derivation dataset were randomly divided into training (70%) and internal testing (30%) set [26] in a way of stratified sampling and repeating it ten times for internal validation. In addition, an independent validation dataset was employed after the model was established to validate the model’s generalization. Because we aimed to develop a simple decision model which could be easier and better applied in clinical practice, parameters in decision tree and random forest followed the default setting in “scikit-learn 1.0.2” package [27], but the number of estimators in random forest was manually set to 3 to simplify the model and decision making. The performance of classification models was evaluated by area under receiver operating characteristic curve (AUC) of each phenotype and micro- and macro-average AUCs. Because the phenotype of new patients in the validation dataset was unknown, the above evaluation indexes were inapplicable. In this case, Kaplan–Meier (KM) estimator and log-rank test were employed to confirm the survival curves of phenotypes [25,28,29,30] classified by the classifier on the validation dataset. Simultaneously, a univariate Cox proportional hazards (CPH) model was performed on each phenotype to explore the phenotype- or risk-level-specific significant risk and protective factors. Finally, the Shapley additive explanations (SHAP) tree explainer [31,32] was elaborated for interpretation of decision making. The detailed system frame was shown in Figure 1.

## 3. Results

### 3.1. Characteristics of Patients

A total of 562 patients with HF were included in the derivation dataset. Overall, the mean ± standard derivation age of patients was 77.8 ± 13.3 years; 257 (45.7%) patients were women. During follow-up of 30.9 ± 13.7 months, all-cause mortality rate was 14.4% (81 patients), where 39 patients died due to cardiovascular diseases. Compared with patients who survived (survival group), patients who died (death group) were significantly older with a heavier burden on kidney function. A comparison of the medical histories between the survival group and the death group did not reveal any significant differences in disease condition, except for ischemic heart disease (IHD; 30.2% vs. 42.0%, *p* = 0.047). Detailed statistical results are summarized in Table S1. Most of characteristics of patients in deviation and validation datasets were comparable, but patients in the validation dataset were more likely to have vascular disease (VD), and higher mitral regurgitation, diastolic blood pressure (DBP), and heart rate (HR), as illustrated in Table S2. In addition, follow up times for the validation dataset (maximum: 31 months) were shorter than the derivation dataset (maximum: 52 months), but it seemed that mortality rate in the validation dataset was slightly higher than the derivation dataset (16.7% vs. 14.4%, *p* = 0.551), this is because almost all the patients in the derivation dataset with a follow-up longer than 31 months survived, which did not contribute to improved mortality rate and, conversely, reduced the overall mortality rate.

### 3.2. Phenotype Exploration and Identification

Patients in the deviation dataset were divided into five clusters, where the optimal number of clusters was determined by dendrogram, scree plot, distortion score and Calinski–Harabasz score together. Details are illustrated in Supplementary Materials, Figure S1. The stability and robustness of the clustering method were validated. As illustrated in Table S3, stability validation showed that there was no significant difference between all the patients and subgroups for each cluster. This indicated that the result of the clustering was reproducible even when conducted on subgroups. Furthermore, the clustering results of agglomerative hierarchical clustering and K-means did not show a significant difference, indicating the determined optimal number of clusters and the distribution of patients in each cluster group were robust.

**Figure 2:**
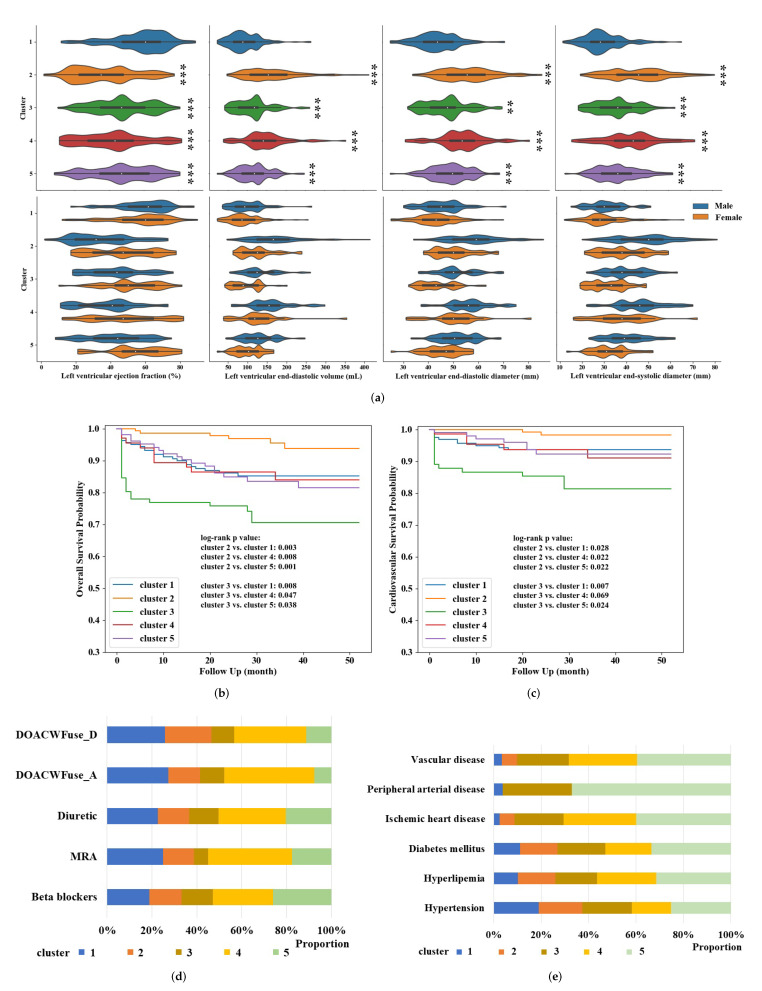
Characteristics of patients clusters 1 (**a**), 2 (**b**), 3 (**c**), 4 (**d**), and 5 (**e**), respectively. The bottom violin plot in (a) showed the characteristics adjusted by gender. “***” in (**a**) indicates *p* < 0.001; “**” in (**b**) indicates *p* < 0.01.

Patients in cluster 1 (n = 160) were highlighted by echocardiographic imaging results. Details are illustrated in Table S4 and visualized by violin plots in Figure 2a; the patients in cluster 1 had higher left ventricular ejection fraction (LVEF; 57.6 ± 16.1%, *p* < 0.001 vs. other clusters), and lower left ventricular end-diastolic volume (93.3 ± 40.7 mL, *p* < 0.001 vs. other clusters), left ventricular end-diastolic diameter (43.8 ± 8.2 mm, *p* < 0.001 vs. clusters 2, 4, 5; *p* = 0.001 vs. cluster 3), and left ventricular end-systolic diameter (30.5 ± 9.1 mm, *p* < 0.001 vs. other clusters). Typically, patients were more likely to be female (68.8%) than in other clusters (*p* < 0.001 vs. clusters 2, 4, 5; *p* = 0.001 vs. cluster 4).

Cluster 2 (n = 142) and cluster 3 (n = 93) had significant differences in mortality risk with other clusters, as shown in Figure 2b,c. Patients in cluster 2 were younger (64.0 ± 14.5 years, *p* < 0.001 vs. other clusters) and had significant higher overall and cardiovascular survival probability than others, which indicated the lowest risk of mortality. Conversely, patients in cluster 3 were oldest (84.7 ± 10.0 years, *p* < 0.05 vs. clusters 2, 4, 5) and had the highest risk of all-cause and cardiovascular mortality (Table S4).

Patients in cluster 4 (n = 66) were more likely to accept pharmacotherapy, as shown in Figure 2d, and illustrated in Table S4, including direct oral anticoagulants or Warfarin (DOACWFuse; 77.3%, *p* < 0.001 vs. other clusters at admission; *p* < 0.001 vs. clusters 2, 3, 5 and *p* = 0.047 vs. cluster 1 at discharge), diuretic (83.3%, *p* < 0.001 vs. clusters 2, 3, 5; *p* = 0.005 vs. cluster 1), and mineralocorticoid receptor antagonist (MRA; 51.5%, *p* < 0.001 vs. clusters 2, 3, 5; *p* = 0.025 vs. cluster 1). In addition, patients in cluster 4 were more likely to experience atrial septal defect after ablation (33.3%, *p* < 0.001 vs. other clusters).

Patients in cluster 5 (n = 103) were more likely to have medical history, as shown in Figure 2e and illustrated in Table S4, such as hypertension (81.6%, *p* < 0.001 vs. clusters 1, 2, 4; *p* = 0.046 vs. cluster 3), hyperlipemia (76.7%, *p* < 0.001 vs. clusters 1, 2, 3; *p* = 0.039 vs. cluster 4), diabetes mellitus (60.2%, *p* < 0.001 vs. clusters 1, 2, 3; *p* = 0.002 vs. cluster 4), IHD (76.7%, *p* < 0.001 vs. clusters 1, 2, 3; *p* = 0.024 vs. cluster 4), peripheral arterial disease (PAD; 33.0%, *p* < 0.001 vs. clusters 1, 2, 4; *p* = 0.004 vs. cluster 3), and VD (81.6%, *p* < 0.001 vs. clusters 1, 2, 3; *p* = 0.002 vs. cluster 4).

**Figure 3:**
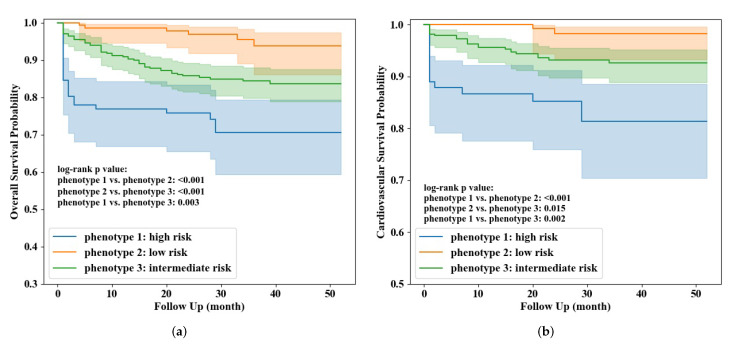
Overall (**a**) and cardiovascular (**b**) survival curves of the three identified phenotypes.

Based on the results of clustering, three patient phenotypes corresponding to stratified mortality risk were identified. Cluster 3 with the highest mortality risk was identified as phenotype 1 (n = 91; all-cause mortality rate: 27.5%; cardiovascular mortality rate: 16.5%), cluster 2 with the lowest mortality risk was phenotype 2 (n = 142, all-cause mortality rate: 4.2%; cardiovascular mortality rate: 1.4%), and phenotype 3 combined clusters 1, 4, and 5 had intermediate risk of mortality (n = 329, all-cause mortality rate: 15.2%; cardiovascular mortality rate: 6.7%). The overall and cardiovascular survival curves with 95% confidence interval (CI) (using Greenwood’s exponential formula) of the three phenotypes were visualized in Figure 3. Log-rank test indicated survival curves among the three phenotypes indeed had significant difference (pairwise comparison *p* < 0.05), and chi-square test confirmed mortality rates in the three phenotypes had significant difference as well (pairwise comparison *p* < 0.05). The hazard ratios of all-cause and cardiovascular mortality between patients in phenotype 1 (high risk) and phenotype 3 (intermediate risk) were 2.08 (95%CI 1.29–3.37, *p* = 0.003) and 2.78 (95%CI 1.44–5.36, *p* = 0.002), respectively; the hazard ratios of all-cause and cardiovascular mortality between patients in phenotype 2 (low risk) and phenotype 3 were 0.26 (95%CI 0.11–0.61, *p* = 0.002) and 0.20 (95%CI 0.05–0.85, *p* = 0.029), respectively.

Phenotype 1, with the highest risk of mortality, was composed of older patients (*p* < 0.001 vs. phenotype 2, *p* = 0.002 vs. phenotype 3) with the lowest estimated glomerular filtration rate (eGFR; *p* < 0.001 vs. phenotype 2, *p* = 0.009 vs. phenotype 3 at admission; *p* < 0.001 vs. other phenotypes at discharge) and creatinine clearance rate (Ccr; *p* < 0.001 vs. other phenotypes), and the highest creatinine (*p* < 0.001 vs. other phenotypes). Phenotype 2 with the lowest risk of mortality was characterized by younger (*p* < 0.001 vs. other phenotypes), the lowest proportion of medical history (*p* < 0.001 vs. other phenotypes) including chronotropic incompetence, IHD, PAD, and VD, and the lowest CHADS${}_{2}$ and CHAD${}_{2}DS_{2}-VAS_{C}$ scores (*p* < 0.001 vs. other phenotypes). Mortality risk and most characteristics in phenotype 3 played a transitional situation between phenotype 1 and 2, as illustrated in Table S5, but phenotype 3 exhibited the highest tricuspid regurgitation (TR; *p* = 0.001 vs. phenotype 1, *p* < 0.001 vs. phenotype 2), and patients in phenotype 3 were more likely to accept pharmacotherapy (*p* < 0.001 vs. other phenotypes) including beta blockers, MRA, diuretic, and direct oral anticoagulants or warfarin (Table S5).

Significant risk and protective factors varied by phenotypes identified by univariate CPH analysis, as illustrated in Table 1. New York Heart Association functional classification (NYHA) of low ADL, DOACWFuse, and HR were phenotype-1-specific significant factors, compared with phenotype 3. Simultaneously, TR, logarithm of N-terminal pro B-type natriuretic peptide (NT-proBNP) (logNT-proBNP), and albumin showed statistical significance only in phenotype 3. Some variables were significant factors in both phenotypes 1 and 3, including eGFR, Ccr, and creatinine, which were parameters related to kidney function. Overall, DOACWFuse, eGFR, Ccr, systolic blood pressure (SBP), DBP, and albumin were protective factors, while NYHA, low ADL, creatinine, HR, TR, and NT-proBNP were risk factors. Because of the rare mortality occurred in phenotype 2 (low risk; mortality: 6/142), we did not conduct the univariate analysis for phenotype 2 in case of the limited power of statistical analysis.

### 3.3. Phenotype Classification

Patients in phenotype 1, 3, and 2 (with high, intermediate, and low risk of mortality, respectively) were labeled as class “0”, “1”, and “2”, respectively. Then, variables performed significant difference among the three phenotypes were used as predictors (Table S5). Based on above univariate CPH analysis, the variable collected at discharge (D) was more likely to be a significant risk or protective factor than the one collected at admission (A). Therefore, for variable collected twice, only the one collected at discharge was used. Then, age, NYHA (≥3), independence in daily life for the elderly with cognitive impairment (IDL) (≥2), LVEF, eGFR, Ccr, creatinine, NT-proBNP, albumin, DBP, and HR at admission were used as predictors. After internal validation, the micro-average AUC, macro-average AUC, AUCs of high, intermediate, low risk were 0.736 ± 0.039, 0.688 ± 0.038, 0.682 ± 0.045, 0.655 ± 0.050, and 0.727 ± 0.051 for the decision tree, respectively; and 0.819 ± 0.016, 0.757 ± 0.024, 0.736 ± 0.038, 0.721 ± 0.036, and 0.815 ± 0.035 for the random forest, respectively. The performance of the two models did not show significant difference.

**Figure 4:**
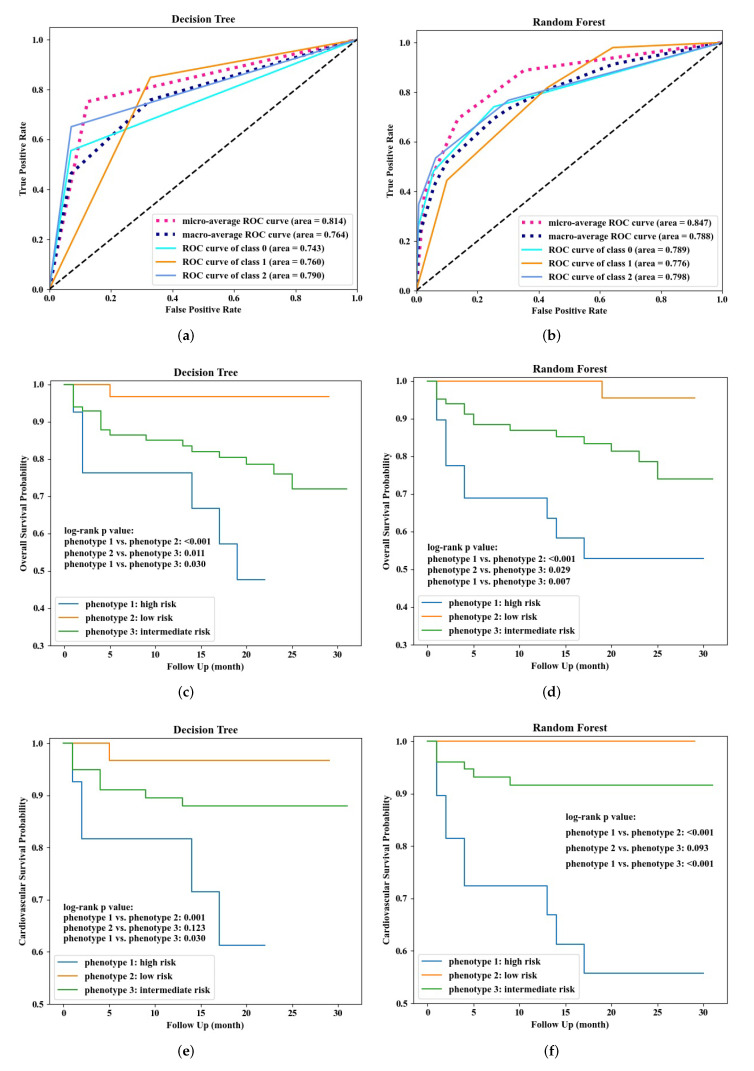
Classification performance of decision tree and random forest. (**a**,**b**) Show ROC curves of the two models on internal validation. (**c**–**f**) Show survival curves of phenotypes classified by decision tree and random forest for patients in the independent validation dataset, respectively.

During the ten experiments, the model performed best was selected as the final classification model. Receiver operating characteristic (ROC) curves of the final used decision tree and random forest models were visualized in Figure 4a,b, respectively. Thereafter, the models classified patients in the validation dataset into three phenotypes, and their survival curves were checked by KM estimator and log-rank test as shown in Figure 4c–f. The overall survival curves of the three classified phenotypes indeed had significant difference, indicating a reliable performance of the models for identifying risk levels of all-cause mortality. However, the two models appeared to have difficulty discriminating between low- and intermediate-risk cardiovascular mortality. However, the two models were particularly good at identifying high-risk patients, and phenotypes classified by random forest had more significant difference than decision tree, indicating a slightly better performance. When validating on complete cases in the validation dataset for sensitivity analysis, similar results were obtained, showing that the classifier was good at identifying high-risk patients, as shown in Figure S2.

**Figure 5:**
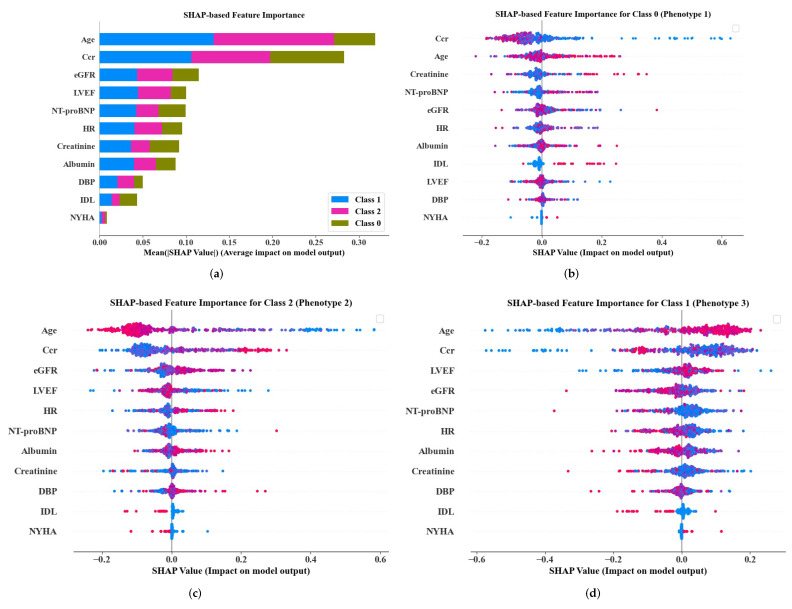
Feature importance for overall (**a**), phenotype 1 (**b**), phenotype 2 (**c**), and phenotype 3 (**d**), interpreted by SHAP.

Based on the results from random forest, feature importance, effects on model output, and decision-making thresholds or cutoff values of important variables were analyzed for transparently interpretation using SHAP. Overall, age, Ccr, eGFR, LVEF, and NT-proBNP were the top five most important variables, as shown in Figure 5a. Figure 5b–d show SHAP summary plots for each phenotype individually, showing the distribution of the impacts each variable had on the model output evaluated by SHAP value. Each point on the summary plot is a SHAP value of a variable for a patient. Variables or predictors were ranked in importance order in the y-axis, and the color indicated the value of the variables from low to high (red—high; blue—low). SHAP values larger than zero in the x-axis push the prediction toward “positive”, for example, in Figure 5b, a higher IDL had a positive SHAP value, so it pushed the model to give a “positive” prediction (class 0: high risk); conversely, a lower Ccr had a positive SHAP value, so it hindered the model to give the “positive” prediction and pushed the model towards “negative” prediction (non class 0). Notably, age, Ccr, NT-proBNP, eGFR, and HR were the top important predictors for all the three phenotypes, where age and Ccr were the top two most important variables.

**Figure 6:**
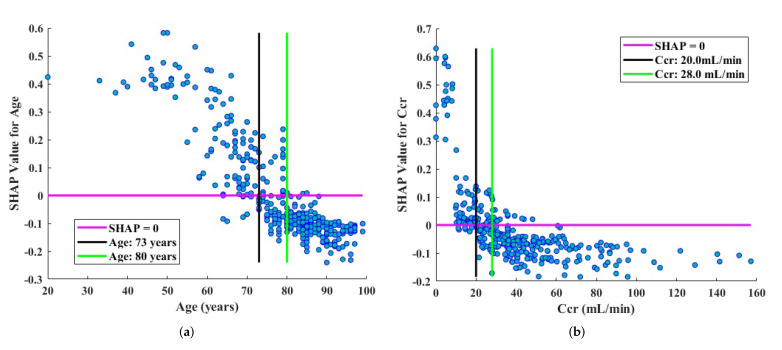
Cutoff values of age (**a**) and Ccr (**b**) and their exact impact on model output.

To further explore the exact effects and cutoff values of the top two most important predictors (age and Ccr), a SHAP dependence plot was enabled. Because age and Ccr were the most important factors, respectively, for phenotype 2 (Figure 5c) and phenotype 1 (Figure 5b), we used the SHAP values of phenotype 2 to analyze age, and Ccr was analyzed using the SHAP values of phenotype 1. Figure 6a implied that age < 73 years was a protective factor which contributed to give the “phenotype 2 or low risk” prediction, and age > 80 years was a risk factor which contributed to give the “non-phenotype 2 or non-low risk” prediction. In detail, hazard ratio of all-cause mortality between patients whose age < 73 years and others (age ≥ 73 years) was 0.28 (95%CI 0.13–0.58, *p* < 0.001) and the hazard ratio between patients older than 80 years and others (age ≤ 80 years) was 2.22 (95%CI 1.40–3.55, *p* < 0.001). In the same way, Figure 6b identified the cutoff values of Ccr were 20.0 mL/min and 28.0 mL/min, and the related hazard ratios were summarized in Table 2.

## 4. Discussion

This study explored and identified phenotypes of patients with HF corresponding to prognosis (risk of mortality during follow-up) from their medical records data during hospitalization and analyzed phenotype-specific characteristics and factors that are significantly associated with mortality. Thereafter, to better apply these findings in clinical settings, such as through identifying the phenotypes of new patients, a phenotype classifier was trained which could aid in making management and treatment plans that take likely prognostic condition into account.

The ML method successfully identified three distinct phenotypes that were characterized by significant difference in prognostic condition, that is, the risk of mortality. The mortality rate in phenotype 1 (high risk) was about twice that of phenotype 3 (intermediate risk) and three times that of phenotype 2 (low risk). Great variability in characteristics and significant risk and protective factors was observed across the phenotypes. Combining unsupervised and supervised ML methods were much of concerned and highlighted in the present study. Determining or identifying the possible prognostic condition for patients by manually viewing their complicated medical records data was irresponsible, subjective, and impossible. Even for cardiologists with prior knowledge and experience, manual prognostic prediction is difficult because of interactions between diseases and complications. However, an unsupervised method could learn from data even without any prior knowledge in hand. Furthermore, the results obtained form the unsupervised method could be used as label information to train a supervised model, since a well-trained model is easier to deploy in clinical applications than an unsupervised model.

It has been reported that mortality of HF increases with advanced age [33]. In this study, patients in the high-risk group were much older than those in the low-risk group (84.7 ± 10.0 vs. 64.0 ± 14.5 years, *p* < 0.001), with a greater proportion of females (47.3% vs. 23.9%, *p* < 0.001) as previously reported by Gustafsson et al. [34]. Based on the CHARM program, Kenchaiah et al. concluded that lower BMI was associated with a greater risk of all-cause and cardiovascular mortality [35], which was consistent with the results of this study. Chronotropic incompetence was common in HF patients and presented a primary cause of severe and symptomatic exercise intolerance, resulting in impaired quality of life [36], and was associated with adverse clinical outcomes, such as all-cause mortality and hospitalization [37,38]. In this study, the prevalence of chronotropic incompetence in the phenotype 1 was about twelvefold greater than phenotype 2. NYHA has been used as a simple descriptor of HF clinically and reported as a strong predictor of mortality with an eightfold increased risk in NYHA III compared with NYHA I [39]. In this study, NYHA class (e.g., III, IV) showed significant difference between phenotype 1 and phenotype 2. Some studies used CHADS${}_{2}$ and CHAD${}_{2}DS_{2}-VAS_{C}$ to predict all-cause mortality [40,41], which were also significantly higher in phenotype 1 than phenotype 2 in this study. Renal insufficiency was confirmed strongly associated with poor outcomes in HF [42], and patients with renal insufficiency were more likely to suffer from drug toxicities, thus preventing them from benefiting from medications [43]. In this study, variables related to kidney function showed significant difference between phenotype 1 and phenotype 2 and were significantly associated with mortality.

In addition, the predictive value of some variables was controversial. Previous studies concluded patients with HF with preserved ejection fraction (HFpEF) had a better prognosis than patients with HF with reduced ejection fraction (HFrEF) [44,45]. By contrast, Abebe et al. found patients with HFpEF or HFrEF had no significant difference in survival [46]. This study showed the phenotype 1 (high risk) had a higher percentage of patients with HFpHF than phenotype 2 (low risk), indicating a different result from above. The possible reason might be that the patients in phenotype 1 were much older than those in phenotype 2 and earlier studies have reported HFpEF was a disease of aging that occurred mainly in older people [47,48,49].

Factors significantly associated with mortality varied by phenotypes or risk levels. Therefore, identifying phenotypes which associated with risk level of mortality was strongly recommended for guiding more precise decision making of treatment strategies and medical resource allocation. It was especially important in determining when to introduce pharmacotherapy or medical devices for patients at high risk [8] or determining advanced discharge for low-risk patients for cost and medical resource saving. Furthermore, univariate CPH analysis suggested greater attention to variables collected at discharge than admission, because most of the significant factors associated with mortality were those collected at discharge.

This study had several limitations. Firstly, the findings presented were explored and identified from a single cohort, which required replication across multiple populations. Secondly, the proposed system could be directly applied for new patients to identify their phenotypes or risk levels of mortality using several easily collected variables, but the exact individual risk score had not been quantified yet. Therefore, the findings in the study were phenotype-specific rather than individual-specific. However, identifying risk groups had considerable clinical significance as well [50,51,52]. Thirdly, this study used mixed cases including HFrEF, patients with HF with with mid-range ejection fraction, and HFpEF. It has reported that the three types differed in treatment and prognostic outcomes [53]; therefore, HF-type-specific analysis is important. Finally, some variables were missing in more than 20% patients, whose data were excluded in this cohort, thus their effects were not included in the presented study.

## 5. Conclusions

ML methods have performed considerable potential to identify distinct clusters and prognostic phenotypes of patients with HF. An unsupervised method, combined with a supervised ML model, was recommended for easier clinical deployment. Furthermore, patients in different mortality risk levels had significant difference in characteristics, risk, or protective factors. Therefore, clinical treatment or management plans are recommended to be formulated or adjusted after careful consideration of prognostic phenotypes or risk levels.

## Figures and Tables

**Table 1:** Univariate analysis for phenotype 1 and phenotype 3.

Variables	Phenotype 1 (High Risk)		Phenotype 3 (Intermediate Risk)	
	Hazard Ratio	*p* Value	Hazard Ratio	*p* Value
NYHA at discharge	3.61 (2.19–5.94)	<0.001	2.31 (1.43-3.75)	<0.001
Low ADL at discharge	2.94 (1.32–6.58)	0.009		- ^1^
DOACWFuse at discharge	0.11 (0.01–0.79)	0.030		-
eGFR at discharge	0.97 (0.95–0.99)	0.009	0.97 (0.95–0.99)	0.002
Ccr at discharge	0.97 (0.93–0.999)	0.045	0.96 (0.93–0.98)	<0.001
Creatinine at discharge	1.15 (1.04–1.27)	0.007	1.78 (1.37–2.30)	<0.001
SBP at admission	0.98 (0.97–0.998)	0.029		-
SBP at discharge	0.95 (0.93–0.96)	<0.001	0.98 (0.97–0.9998)	0.047
DBP at discharge	0.95 (0.92–0.99)	0.006		-
HR at discharge	1.02 (1.002–1.05)	0.035		-
TR		-	1.29 (1.03–1.62)	0.025
logNT-proBNP		-	2.64 (1.45–4.79)	0.001
Albumin		-	0.55 (0.31–0.97)	0.039

^1^ Not statistically significant.

**Table 2:** Hazard ratio between two patient groups classified by cutoff values of the top two most important predictors.

Variables and Cutoff Values	Hazard Ratio	*p* Value
Age < 73 years	0.28 (0.13–0.58)	<0.001
Age > 80 years	2.22 (1.40–3.55)	<0.001
Ccr at discharge < 20 mL/min	3.63 (2.34–5.63)	<0.001
Ccr at discharge > 28 mL/min	0.35 (0.22–0.55)	<0.001

## Data Availability

The data used in this article are not readily available because the restrictions of the Institutional Review Board. Requests to access the datasets should be directed to corresponding authors.

## Supplementary Materials

The following are available at https://www.mdpi.com/article/10.3390/life12060776/s1, Figure S1: Optimal number of clusters (k = 5) determined by dendrogram (**a**), scree plot (**b**) and elbow method based on distortion score (**c**) and calinski harabasz score (**d**), Figure S2: Survival curves of phenotypes classified by random forest for complete cases in validation dataset, Table S1: Characteristics of patients with heart failure in derivation dataset, Table S2: Comparison between patients in derivation and validation datasets, Table S3: Validation of stability and robustness of clustering, Table S4: Characteristics of clusters, Table S5: Characteristics of phenotypes.

## Abbreviations

The following abbreviations are used in this manuscript:

ADL	activities of daily living
AUC	area under receiver operating characteristic curve
BMI	body mass index
Ccr	creatinine clearance rate
CI	confidence interval
CPH	Cox proportional hazards
DBP	diastolic blood pressure
DOACWFuse	direct oral anticoagulants or warfarin used
DPC	diagnosis procedure combination
eGFR	estimated glomerular filtration rate
HF	heart failure
HFpEF	heart failure with preserved ejection fraction
HFrEF	heart failure with reduced ejection fraction
HR	heart rate
IDL	independence in daily life for the elderly with cognitive impairment
IHD	ischemic heart disease
KM	Kaplan–Meier
LVEF	left ventricular ejection fraction
ML	machine learning
MRA	mineralocorticoid receptor antagonist
NT-proBNP	N-terminal pro B-type natriuretic peptide
NYHA	New York Heart Association
PAD	peripheral arterial disease
ROC	receiver operating characteristic curve
SHAP	Shapley additive explanations
TR	tricuspid regurgitation
VD	vascular disease
