# Supplementary material for: Exploring and Identifying Prognostic Phenotypes of Patients with Heart Failure Guided by Explainable Machine Learning
Source: Life (Basel). 2022 May 24;12(6):776. doi: 10.3390/life12060776 (PMC9224610; doi:10.3390/life12060776)
Supplement: Supplementary file 1 [file life-12-00776-s001.zip › life-1723112-supplementary.pdf]

Table S1. Characteristics of patients with heart failure in derivation dataset

Variables	Total (n=562)	All-cause mortality (n = 81)	Survive (n = 481)	P value
Follow Up (month)	30.9±13.7	9.9±10.3	34.4±10.8	<0.001
Cardiovascular mortality	39 (6.9)	39 (48.15)	0 (0)	NA
Age (years)	77.8±13.3	82.8±9.0	77.0±13.7	<0.001
Female	257 (45.7)	33 (40.7)	224 (46.6)	0.393
Length of stay (day)	19.3±15.7	19.3±13.2	19.3±16.1	0.467
Prior admission times	2.4±2.1	2.9±2.3	2.3±2.1	0.002
Weight (kg)	54.4±14.8	51.7±12.3	54.9±15.2	0.041
Body mass index (kg/m^2)	21.7±4.4	21.0±4.0	21.8±4.4	0.190
Hypertension	363 (64.6)	56 (69.1)	307 (63.8)	0.424
Hyperlipemia	252 (44.8)	42 (51.9)	210 (43.7)	0.211
Diabetes mellitus	190 (33.8)	27 (33.3)	163 (33.9)	0.977
Chronotropic incompetence	88 (15.7)	14 (17.3)	74 (15.4)	0.787
Ischemic heart disease	179 (31.9)	34 (42.0)	145 (30.2)	0.047
Peripheral arterial disease	50 (8.9)	10 (12.4)	40 (8.3)	0.333
Atrial fibrillation	303 (53.9)	43 (53.1)	260 (54.1)	0.967
Vascular disease	194 (34.5)	34 (42.0)	160 (33.3)	0.162
New York Heart Association (NYHA) at admission				0.469
1	24 (4.3)	4 (4.9)	20 (4.2)	
2	147 (26.2)	20 (24.7)	127 (26.4)	
3	281 (50.0)	37 (45.7)	244 (50.7)	
4	110 (19.6)	20 (24.7)	90 (18.7)	
NYHA at discharge				<0.001
1	66 (11.7)	5 (6.2)	61 (12.7)	
2	426 (75.8)	48 (59.3)	378 (78.6)	
3	54 (9.6)	18 (22.2)	36 (7.5)	
4	16 (2.9)	10 (12.4)	6 (1.3)	
Frail (frailty class≥3)	525 (93.4)	79 (97.5)	446 (92.7)	0.170
$CHADS_2$	3.0±1.2	3.2±1.2	2.9±1.2	0.044
CHA_2DS_2-VAS_C	5.2±1.8	5.6±1.7	5.1±1.8	0.034
Independence in daily life for the elderly with cognitive impairment				<0.001
0	425 (75.6)	48 (59.3)	377 (78.4)	
1	99 (17.6)	23 (28.4)	76 (15.8)	
2	31 (5.5)	9 (11.1)	22 (4.6)	
3	3 (0.5)	0 (0)	3 (0.6)	
4	4 (0.7)	1 (1.2)	3 (0.6)	
Low ADL at admission	72 (12.8)	17 (21.0)	55 (11.4)	0.028
Low ADL at discharge	31 (5.5)	14 (17.3)	17 (3.5)	<0.001
Left ventricular (LV) ejection fraction (%)	47.2±18.8	46.5±19.0	47.4±18.8	0.700
HFrEF	197 (35.0)	29 (35.8)	168 (34.9)	

**HFmrEF**	**114 (20.3)**	**18 (22.2)**	**96 (20.0)**	
**HFpEF**	**251 (44.7)**	**34 (42.0)**	**217 (45.1)**	
**LV end-diastolic volume (mL)**	**124.8±58.7**	**131.4±73.7**	**123.7±55.9**	**0.845**
**LV end-diastolic dimension (mm)**	**49.7±10.2**	**50.5±12.2**	**49.6±9.8**	**0.506**
**LV end-systolic diameter (mm)**	**38.1±12.3**	**39.2±14.3**	**37.9±12.0**	**0.678**
**Left atrial diameter (mm)**	**45.0±8.4**	**46.0±8.8**	**44.9±8.3**	**0.303**
**Mitral regurgitation**	**1.5±1.1**	**1.8±1.1**	**1.5±1.1**	**0.030**
**Tricuspid regurgitation**	**1.3±1.1**	**1.6±1.2**	**1.3±1.0**	**0.034**
**RVSP (mmHg)**	**29.2±10.3**	**31.0±11.1**	**28.9±10.2**	**0.053**
**Atrial septal defect after ablation**	**30 (5.3)**	**4 (4.9)**	**26 (5.4)**	**0.925**
**Aortic valve replacement**	**27 (4.8)**	**5 (6.2)**	**22 (4.6)**	**0.733**
**Hemodialysis**	**21 (3.7)**	**5 (6.2)**	**16 (3.3)**	**0.351**
**ACEi/ARB**	**268 (47.7)**	**51 (63.0)**	**217 (45.1)**	**0.004**
**Beta blockers**	**286 (50.9)**	**48 (59.3)**	**238 (49.5)**	**0.131**
**Mineralocorticoid receptor antagonist**	**149 (26.5)**	**28 (34.6)**	**121 (25.2)**	**0.101**
**Diuretic**	**302 (53.7)**	**49 (60.5)**	**253 (52.6)**	**0.231**
**DOACWFuse at admission**	**208 (37.0)**	**34 (42.0)**	**174 (36.2)**	**0.381**
**DOACWFuse at discharge**	**272 (48.4)**	**34 (42.0)**	**238 (49.5)**	**0.919**
**eGFR at admission (mL/min/1.73m^2)**	**42.8±21.1**	**34.7±21.4**	**44.2±20.8**	**<0.001**
**eGFR at discharge (mL/min/1.73m^2)**	**40.8±21.2**	**29.2±18.9**	**42.7±21.0**	**<0.001**
**Creatinine clearance rate (Ccr) at admission (mL/min)**	**40.3±26.9**	**28.0±17.6**	**42.4±27.6**	**<0.001**
**Ccr at discharge (mL/min)**	**37.9±24.8**	**24.6±16.4**	**40.1±25.3**	**<0.001**
**Creatinine (Cr) at admission (mg/dL)**	**1.5±1.3**	**1.9±1.5**	**1.4±1.2**	**<0.001**
**Cr at discharge (mg/dL)**	**1.6±1.5**	**2.5±2.5**	**1.4±1.2**	**<0.001**
**Albumin (g/dL)**	**3.4±0.5**	**3.3±0.5**	**3.4±0.5**	**0.007**
**Total protein (g/dL)**	**7.0±0.6**	**7.0±0.6**	**7.0±0.6**	**0.592**
**C-reactive protein (mg/dL)**	**2.3±4.1**	**2.6±3.6**	**2.3±4.2**	**0.041**
**Hemoglobin (g/dL)**	**11.9±2.4**	**11.3±2.1**	**12.0±2.4**	**0.008**
**Systolic blood pressure (SBP) at admission (mmHg)**	**132.1±25.9**	**128.2±31.9**	**132.7±24.7**	**0.224**
**SBP at discharge (mmHg)**	**112.2±18.1**	**104.9±24.7**	**113.4±16.4**	**0.003**
**Diastolic blood pressure (DBP) at admission (mmHg)**	**72.8±32.2**	**76.9±73.4**	**72.1±17.7**	**0.272**
**DBP at discharge (mmHg)**	**59.3±10.3**	**55.9±12.8**	**59.9±9.7**	**0.008**
**Heart rate at admission (bmp)**	**85.3±23.1**	**83.5±19.4**	**85.6±23.7**	**0.394**

**Heart rate at discharge (bmp)**	**70.2±13.9**	**73.2±18.6**	**69.7±12.9**	**0.104**

ACEi (Angiotensin-converting enzyme inhibitor); ADL (activities of daily living); ARB (Angiotensin receptor blocker); $CHADS_2$ (congestive heart failure: 1 point; hypertension: 1 point, age ≥ 75 years: 1 point; diabetes mellitus: 1 point; prior stroke or TIA or thromboembolism: 2 points); $CHAD_2DS_2\text{-}VAS_C$ score (congestive heart failure: 1 point, hypertension: 1 point; age ≥ 75 years: 2 points; diabetes mellitus: 1 point; prior stroke or TIA or thromboembolism: 2 points; vascular disease: 1 point; age 65-74 years: 1 point; sex category: 1 point); DOACWFuse (Direct oral anticoagulants or Warfarin usage); eGFR (estimated glomerular filtration rate); HFmrEF (heart failure with mid-range ejection fraction); HFpEF (heart failure with preserved ejection fraction); HFrEF (heart failure with reduced ejection fraction); NYHA (New York Heart Association); RVSP (Right ventricular systolic pressure)

Table S2. Comparison between patients in derivation and validation datasets

Variables	Derivation (n=562)	Validation (n=168)	P value
Follow Up (month)	30.9±13.7	13.6±10.2	<0.001
All-cause mortality	81 (14.4)	28 (16.7)	0.551
Cardiovascular mortality	39 (6.9)	17 (10.1)	0.233
Age (years)	77.8±13.3	78.0±12.8	0.927
Female	257 (45.7)	74 (44.1)	0.767
Length of stay (day)	19.3±15.7	18.5±13.3	0.836
Weight (kg)	54.4±14.8	52.8±12.0	0.142
Body mass index (kg/m^2)	21.7±4.4	20.8±3.7	0.017
Hypertension	363 (64.6)	107 (63.7)	0.903
Hyperlipemia	252 (44.8)	73 (43.5)	0.819
Diabetes mellitus	190 (33.8)	53 (31.6)	0.651
Chronotropic incompetence	88 (15.7)	32 (19.1)	0.357
Ischemic heart disease	179 (31.9)	64 (38.1)	0.157
Peripheral arterial disease	50 (8.9)	22 (13.1)	0.146
Atrial fibrillation	303 (53.9)	94 (56.0)	0.706
Vascular disease	194 (34.5)	74 (44.1)	0.031
New York Heart Association (NYHA) at admission			0.080
1	24 (4.3)	17 (10.1)	
2	147 (26.2)	42 (25.0)	
3	281 (50.0)	83 (49.4)	
4	110 (19.6)	26 (15.5)	
NYHA at discharge			0.005
1	66 (11.7)	38 (22.6)	
2	426 (75.8)	112 (66.7)	
3	54 (9.6)	12 (7.1)	
4	16 (2.9)	6 (3.6)	
Frail (frailty class≥3)	525 (93.4)	151 (89.9)	0.171
$CHADS_2$	3.0±1.2	3.0±1.2	0.800
CHA_2DS_2-VAS_C	5.2±1.8	4.7±1.6	0.002
Independence in daily life for the elderly with cognitive impairment			0.627
0	425 (75.6)	134 (79.8)	
1	99 (17.6)	11 (6.6)	
2	31 (5.5)	10 (6.0)	
3	3 (0.5)	7 (4.2)	
4	4 (0.7)	6 (3.6)	
Low activities of daily life (ADL) at admission	72 (12.8)	18 (10.7)	0.554
Low ADL at discharge	31 (5.5)	8 (4.8)	0.853
Left ventricular (LV) ejection fraction (%)	47.2±18.8	45.1±18.6	0.192
HFrEF	197 (35.0)	67 (39.9)	
HFmrEF	114 (20.3)	30 (17.8)	

**HFpEF**	**251 (44.7)**	**71 (42.3)**	
**LV end-diastolic volume (mL)**	**124.8±58.7**	**121.0±50.3**	**0.715**
**LV end-diastolic dimension (mm)**	**49.7±10.2**	**49.1±9.0**	**0.433**
**LV end-systolic diameter (mm)**	**38.1±12.3**	**38.1±11.2**	**0.777**
**Left atrial diameter (mm)**	**45.0±8.4**	**44.3±8.6**	**0.356**
**Mitral regurgitation**	**1.5±1.1**	**1.7±1.0**	**0.014**
**Tricuspid regurgitation**	**1.3±1.1**	**1.3±1.0**	**0.365**
**Right ventricular systolic pressure (mmHg)**	**29.2±10.3**	**27.3±10.2**	**0.034**
**Atrial septal defect after ablation**	**30 (5.3)**	**13 (7.7)**	**0.331**
**Aortic valve replacement**	**27 (4.8)**	**10 (6.0)**	**0.693**
**Hemodialysis**	**21 (3.7)**	**5 (3.0)**	**0.819**
**Angiotensin-converting enzyme inhibitor/Angiotensin receptor blocker**	**268 (47.7)**	**64 (38.1)**	**0.036**
**Beta blockers**	**286 (50.9)**	**85 (50.6)**	**1**
**Mineralocorticoid receptor antagonist**	**149 (26.5)**	**37 (22.0)**	**0.284**
**Diuretic**	**302 (53.7)**	**73 (43.5)**	**0.024**
**Direct oral anticoagulants/Warfarin usage at admission**	**208 (37.0)**	**73 (43.5)**	**0.157**
**Direct oral anticoagulants/Warfarin usage at discharge**	**272 (48.4)**	**90 (53.6)**	**0.276**
**Estimated glomerular filtration rate (eGFR) at admission (mL/min/1.73m^2)**	**42.8±21.1**	**44.1±19.6**	**0.458**
**eGFR at discharge (mL/min/1.73m^2)**	**40.8±21.2**	**47.4±61.3**	**0.197**
**Creatinine clearance rate (Ccr) at admission (mL/min)**	**40.3±26.9**	**36.6±20.2**	**0.382**
**Ccr at discharge (mL/min)**	**37.9±24.8**	**38.1±34.8**	**0.669**
**Creatinine (Cr) at admission (mg/dL)**	**1.5±1.3**	**1.4±1.0**	**0.257**
**Cr at discharge (mg/dL)**	**1.6±1.5**	**1.5±1.3**	**0.063**
**Albumin (g/dL)**	**3.4±0.5**	**3.4±0.5**	**0.491**
**Total protein (g/dL)**	**7.0±0.6**	**6.9±0.7**	**0.094**
**C-reactive protein (mg/dL)**	**2.3±4.1**	**2.1±4.2**	**0.121**
**Hemoglobin (g/dL)**	**11.9±2.4**	**12.1±2.3**	**0.316**
**Systolic blood pressure (SBP) at admission (mmHg)**	**132.1±25.9**	**139.0±32.8**	**0.066**
**SBP at discharge (mmHg)**	**112.2±18.1**	**119.8±81.0**	**0.422**
**Diastolic blood pressure (DBP) at admission (mmHg)**	**72.8±32.2**	**80.6±24.1**	**<0.001**
**DBP at discharge (mmHg)**	**59.3±10.3**	**61.5±10.4**	**0.017**
**Heart rate at admission (bmp)**	**85.3±23.1**	**91.7±27.3**	**0.006**
**Heart rate at discharge (bmp)**	**70.2±13.9**	**70.9±14.6**	**0.592**

The optimal number of clusters

The patients in derivation dataset seemed to be divided into two clusters (one in green, the other in red) based on dendrogram shown in **Figure S1(a)**, or each of which could be further divided into two groups, to obtain four clusters. However, it was unreliable to determine the optimal number of clusters, i.e., "k" by visually inspecting the dendrogram alone. Viewing in the scree plot shown in **Figure S1(b)**, there was a turning point where distance equals 12.7 approximately. Before this turning point, the distance reduced greatly for each increment in the number of clusters. After this point, while the number of clusters increased the distance reduced asymptotically, suggesting "k" should correspond to this turning point. In this case, a horizontal line (black dotted line) passing over the dendrogram in **Figure S1(a)** at a distance equaling 12.7 divided the clusters at 5 points, or 6 or even 7 points. This imprecision arose because there were two red groups that just touched the horizontal line. Therefore, the distortion score and Calinski–Harabasz score were used for further confirmation. As shown in **Figure S1(c, d)**, both scores determined that the optimal number of clusters was 5 (k = 5).

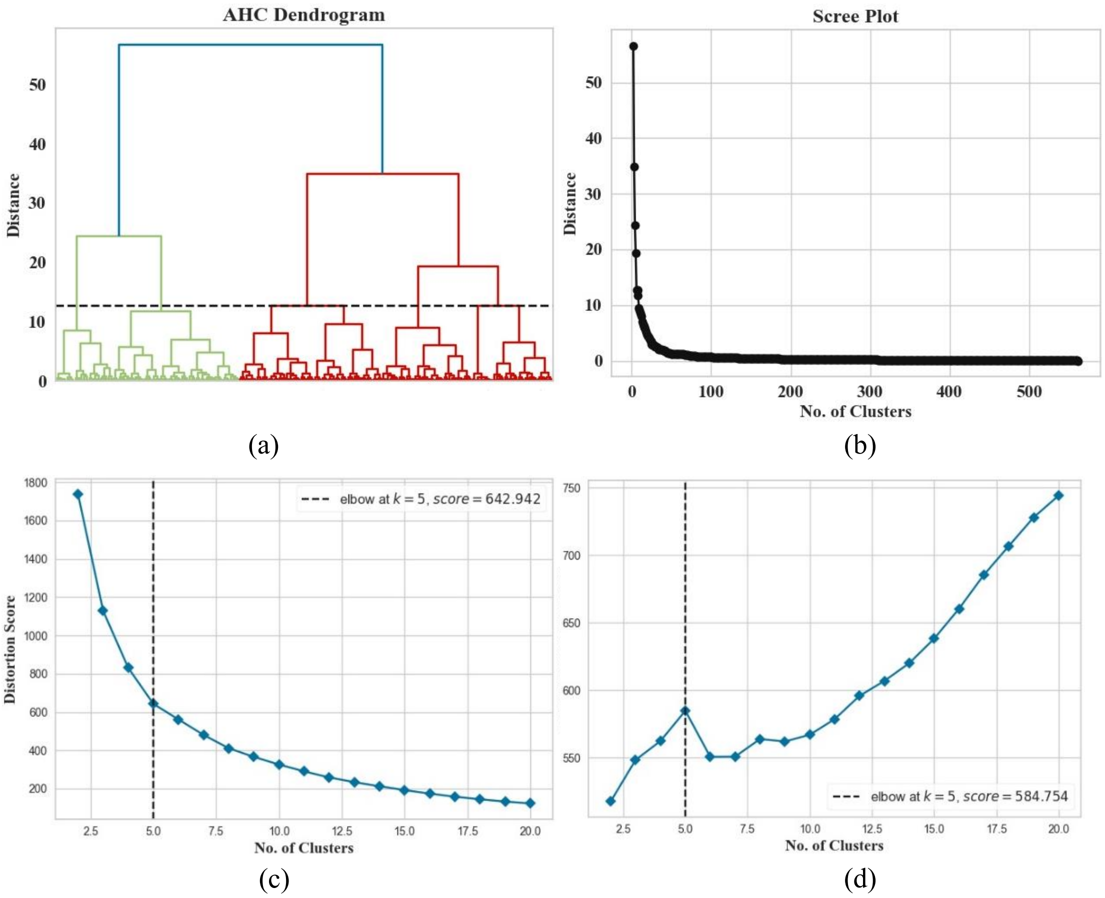

Figure S1. Optimal number of clusters (k = 5) determined by dendrogram (**a**), scree plot (**b**) and elbow method based on distortion score (**c**) and calinski harabasz score (**d**).

Table S3 Validation of stability and robustness of clustering

	Cluster1	Cluster2	Cluster3	Cluster4	Cluster5	Total
Number of patients	160 (28.5)	142 (25.3)	91 (16.2)	66 (11.7)	103 (18.3)	562
Validation of stability						
Subgroup1	29 (25.7)	25 (22.1)	20 (17.7)	17 (15.0)	22 (19.5)	113
P value	0.623	0.557	0.799	0.413	0.879	
Subgroup2	30 (26.5)	29 (25.7)	22 (19.5)	8 (7.1)	24 (21.2)	113
P value	0.764	1	0.476	0.200	0.555	
Subgroup3	37 (32.7)	27 (23.9)	18 (15.9)	8 (7.1)	22 (19.5)	112
P value	0.392	0.889	1	0.209	0.846	
Subgroup4	35 (31.0)	28 (24.8)	15 (13.3)	13 (11.5)	21 (18.6)	112
P value	0.632	1	0.548	1	1	
Subgroup5	28 (24.8)	23 (20.4)	21 (18.6)	17 (15.0)	23 (20.4)	112
P value	0.527	0.346	0.600	0.394	0.678	
Validation of robustness						
K-means	153 (27.2)	138 (24.6)	98 (17.4)	68 (12.1)	23 (20.4)	562
P value	0.690	0.836	0.632	0.927	0.678	

Table S4 Characteristics of clusters

Variables	Cluster1 (n = 160)	cluster2 (n = 142)	Cluster3 (n = 91)	Cluster4 (n = 66)	Cluster5 (n = 103)
All-cause mortality	23 (14.4)	6 (4.2)	25 (27.5)	10 (15.2)	17 (16.5)
Follow Up (month)	30.9±13.9	32.9±11.0	25.8±16.2	32.2±13.9	31.6±13.6
Cardiovascular mortality	10 (6.3)	2 (1.4)	15 (16.5)	5 (7.6)	7 (6.8)
Age (years)	83.4±7.6	64.0±14.5	84.7±10.0	76.8±9.4	82.5±7.7
Female	110 (68.8)	34 (23.9)	43 (47.3)	27 (40.9)	43 (41.8)
Length of stay	21.6±18.8	15.0±9.6	24.0±20.1	17.3±12.7	18.3±12.6
Admission times	2.1±1.7	1.5±1.0	2.3±2.1	3.7±3.0	3.2±2.6
Weight (kg)	46.5±10.5	66.6±15.0	49.8±12.9	55.6±15.2	53.4±10.2
BMI (kg/m^2)	20.0±3.8	24.3±4.6	20.4±4.0	21.9±4.3	21.7±3.7
Hypertension	98 (61.3)	84 (59.2)	62 (68.1)	35 (53.0)	84 (81.6)
Hyperlipemia	40 (25.0)	54 (38.0)	39 (42.9)	40 (60.6)	79 (76.7)
Diabetes mellitus	32 (20.0)	40 (28.2)	33 (36.3)	23 (34.9)	62 (60.2)
Chronotropic incompetence	31 (19.4)	3 (2.1)	22 (24.2)	7 (10.6)	25 (24.3)
Ischemic heart disease	8 (5.0)	17 (12.0)	36 (39.6)	39 (59.1)	79 (76.7)
Peripheral arterial disease	3 (1.9)	0 (0)	13 (14.3)	0 (0)	34 (33.0)
Atrial fibrillation	109 (68.1)	69 (48.6)	40 (44.0)	50 (75.8)	35 (34.0)
Vascular disease	11 (6.9)	19 (13.4)	41 (45.1)	39 (59.1)	84 (81.6)
New York Heart Association (NYHA) at admission					
1	7 (4.4)	9 (6.3)	5 (5.5)	2 (3.0)	1 (1.0)
2	48 (30.0)	37 (26.1)	11 (12.1)	22 (33.3)	29 (28.2)
3	83 (51.9)	77 (54.2)	39 (42.9)	32 (48.5)	50 (48.5)
4	22 (13.8)	19 (13.4)	36 (39.6)	10 (15.2)	23 (22.3)
NYHA at discharge					
1	9 (5.6)	41 (28.9)	8 (8.8)	5 (7.6)	3 (2.9)
2	129 (80.6)	99 (69.7)	55 (60.4)	55 (83.3)	88 (85.4)
3	19 (11.9)	0 (0)	19 (20.9)	5 (7.6)	11 (10.7)
4	3 (1.9)	2 (1.4)	9 (9.9)	1 (1.5)	1 (1.0)
Frail (frailty≥3)	158 (98.8)	107 (75.4)	91 (100)	66 (100)	103 (100)
$CHADS_2$	3.1±1.2	2.2±0.9	3.3±1.2	2.7±0.9	3.8±1.0
$CHAD_2DS_2$-VASc	5.4±1.6	3.5±1.4	5.8±1.4	5.1±1.3	6.6±1.4
Independence in daily life for the elderly with dementia					
0	113 (70.6)	136 (95.8)	41 (45.1)	53 (80.3)	82 (79.6)
1	37 (23.1)	6 (4.2)	27 (29.7)	12 (18.2)	17 (16.5)
2	6 (3.8)	0 (0)	20 (22.0)	1 (1.5)	4 (3.9)
3	2 (1.3)	0 (0)	1 (1.1)	0 (0)	0 (0)
4	2 (1.3)	0 (0)	4 (2.2)	0 (0)	0 (0)
Low activities of daily life (ADL) at admission	6 (3.8)	5 (3.5)	53 (58.2)	2 (3.0)	6 (5.8)
Low ADL at discharge	3 (1.9)	2 (1.4)	25 (27.5)	0 (0)	1 (1.0)
Left ventricular (LV) ejection	57.6±16.1	37.7±18.0	47.2±16.1	42.3±18.9	47.4±17.9

fraction (EF) (%)					
LV end-diastolic volume (mL)	93.3±40.7	161.9±69.7	115.5±45.3	147.3±58.9	116.6±41.3
LV end-diastolic diameter (mm)	43.8±8.2	56.3±10.6	47.7±8.5	53.8±9.6	48.8±7.8
LV end-systolic diameter (mm)	30.5±9.1	46.2±13.2	36.4±9.5	42.8±12.2	37.1±9.7
Left atrial diameter (mm)	45.1±10.5	45.9±7.2	43.0±7.3	48.9±8.0	42.9±6.3
Mitral regurgitation	1.5±1.1	1.5±1.0	1.5±1.0	1.9±1.2	1.4±0.9
Tricuspid regurgitation	1.8±1.2	1.0±0.8	1.1±0.8	1.5±1.1	1.0±0.8
Right ventricular systolic pressure (mmHg)	30.8±11.6	25.9±8.8	28.7±9.0	31.1±11.3	30.2±9.8
Atrial septal defect after ablation	3 (1.9)	3 (2.1)	2 (2.2)	22 (33.3)	0 (0)
Aortic valve replacement	17 (10.6)	2 (1.4)	3 (3.3)	0 (0)	5 (4.9)
Hemodialysis	0 (0)	0 (0)	21 (23.1)	0 (0)	0 (0)
Angiotensin-converting enzyme inhibitor /Angiotensin receptor blocker	79 (49.4)	52 (36.6)	37 (40.7)	35 (53.0)	65 (63.1)
Beta blockers	80 (50.0)	54 (38.0)	34 (37.4)	47 (71.2)	71 (68.9)
Mineralocorticoid receptor antagonist	55 (34.4)	27 (19.0)	8 (8.8)	34 (51.5)	25 (24.3)
Diuretic	101 (63.1)	55 (38.7)	33 (36.3)	55 (83.3)	58 (56.3)
Direct oral anticoagulants/Warfarin used at admission	84 (52.5)	39 (27.5)	19 (20.9)	51 (77.3)	15 (14.6)
Direct oral anticoagulants/Warfarin used at discharge	100 (62.5)	71 (50.0)	22 (24.2)	51 (77.3)	28 (27.2)
Estimated glomerular filtration rate (eGFR) at admission (mL/min/1.73m^2)	41.9±19.9	54.5±17.7	32.6±27.1	40.6±15.4	38.6±17.3
eGFR at discharge (mL/min/1.73m^2)	41.1±22.1	50.0±16.4	32.7±29.0	37.9±15.7	36.5±15.7
Creatinine clearance rate (Ccr) at admission (mL/min)	31.1±15.0	69.7±30.8	19.8±13.1	39.2±17.6	32.9±13.8
Ccr at discharge (mL/min)	30.7±15.4	63.0±30.3	20.2±13.3	35.3±14.2	31.4±12.8
Creatinine at admission (mg/dL)	1.2±0.6	1.1±0.4	2.7±2.5	1.3±0.6	1.4±0.7
Creatinine at discharge (mg/dL)	1.3±0.6	1.2±0.5	3.0±3.1	1.5±0.7	1.5±0.7
Albumin (g/dL)	3.3±0.5	3.6±0.4	3.2±0.5	3.6±0.4	3.4±0.5
Total protein (g/dL)	6.9±0.6	7.0±0.6	7.0±0.7	7.1±0.6	7.0±0.6
C-reactive protein (mg/dL)	2.6±4.9	1.4±2.5	4.1±5.5	1.6±2.6	2.0±3.1

**Hemoglobin (g/dL)**	**11.1±2.0**	**13.6±2.5**	**11.0±2.1**	**12.1±2.0**	**11.4±2.0**
**Systolic blood pressure (SBP) at admission (mmHg)**	**127.7±24.5**	**131.4±25.9**	**137.5±27.1**	**127.1±23.4**	**138.1±26.6**
**SBP at discharge (mmHg)**	**110.7±18.3**	**109.8±16.2**	**110.0±20.4**	**109.2±11.8**	**121.7±18.4**
**Diastolic blood pressure (DBP) at admission (mmHg)**	**67.7±14.1**	**76.6±20.1**	**79.7±69.0**	**68.7±14.3**	**72.1±20.0**
**DBP at discharge (mmHg)**	**57.7±11.1**	**62.0±9.3**	**56.0±11.1**	**60.8±8.3**	**60.2±9.4**
**Heart rate at admission (bmp)**	**80.0±21.9**	**94.6±26.9**	**86.9±21.0**	**85.8±19.1**	**78.8±18.9**
**Heart rate at discharge (bmp)**	**73.2±12.9**	**69.8±13.1**	**69.7±16.8**	**71.3±13.8**	**65.9±12.5**

Table S5 Characteristics of phenotypes

Variables	Phenotype 1 (n = 91)	Phenotype 2 (n = 142)	Phenotype 3 (n = 329)	1 vs. 2	1 vs. 3	2 vs. 3
Risk level	High	Low	Intermediate			
All-cause mortality	25 (27.5)	6 (4.2)	50 (15.2)	<0.001	0.011	0.001
Follow Up (month)	25.8±16.2	32.9±11.0	31.4±13.8	0.003	0.005	0.591
Cardiovascular mortality	15 (16.5)	2 (1.4)	22 (6.7)	<0.001	0.007	0.031
Age (years)	84.7±10.0	64.0±14.5	81.2±8.4	<0.001	0.002	<0.001
Female	43 (47.3)	34 (23.9)	180 (54.7)	<0.001	0.253	<0.001
Length of stay (day)	24.0±20.1	15.0±9.6	19.9±16.1	<0.001	0.103	0.005
Prior admission times	2.3±2.1	1.5±1.0	2.8±2.4	<0.001	0.023	<0.001
Weight (kg)	49.8±12.9	66.6±15.0	50.5±12.1	<0.001	0.646	<0.001
BMI (kg/m^2)	20.4±4.0	24.3±4.6	20.9±3.9	<0.001	0.339	<0.001
Hypertension	62 (68.1)	84 (59.2)	217 (66.0)	0.214	0.792	0.192
Hyperlipemia	39 (42.9)	54 (38.0)	159 (48.3)	0.550	0.420	0.050
Diabetes mellitus	33 (36.3)	40 (28.2)	117 (35.6)	0.248	1	0.146
Chronotropic incompetence	22 (24.2)	3 (2.1)	63 (19.2)	<0.001	0.363	<0.001
Ischemic heart disease	36 (39.6)	17 (12.0)	126 (38.3)	<0.001	0.923	<0.001
Peripheral arterial disease	13 (14.3)	0 (0)	37 (11.3)	<0.001	0.542	<0.001
Atrial fibrillation	40 (44.0)	69 (48.6)	194 (59.0)	0.577	0.015	0.048
Vascular disease	41 (45.1)	19 (13.4)	134 (40.7)	<0.001	0.535	<0.001
New York Heart Association (NYHA) at admission				<0.001	<0.001	0.607
1	5 (5.5)	9 (6.3)	10 (3.0)			
2	11 (12.1)	37 (26.1)	99 (30.1)			
3	39 (42.9)	77 (54.2)	165 (50.2)			
4	36 (39.6)	19 (13.4)	55 (16.7)			
NYHA at discharge (NYHA_D)				<0.001	0.002	<0.001
1	8 (8.8)	41 (28.9)	17 (5.2)			
2	55 (60.4)	99 (69.7)	272 (82.7)			
3	19 (20.9)	0 (0)	35 (10.6)			
4	9 (9.9)	2 (1.4)	5 (1.5)			
NYHA_D > 2	28 (30.8)	2 (1.4)	40 (12.2)	<0.001	<0.001	<0.001
Frail (frailty class≥3)	91 (100)	107 (75.4)	327 (99.4)	<0.001	1	<0.001
$CHADS_2$	3.3±1.2	2.2±0.9	3.2±1.1	<0.001	0.529	<0.001
$CHAD_2DS_2$-VASc	5.8±1.4	3.5±1.4	5.7±1.6	<0.001	0.529	<0.001
Independence in daily life for the elderly with dementia				<0.001	<0.001	<0.001
0	41 (45.1)	136 (95.8)	248 (75.4)			
1	27 (29.7)	6 (4.2)	66 (20.1)			
2	20 (22.0)	0 (0)	11 (3.3)			
3	1 (1.1)	0 (0)	2 (0.6)			
4	4 (2.2)	0 (0)	2 (0.6)			
IDL > 1	25 (27.5)	0 (0)	15 (4.6)	<0.001	<0.001	0.007

**Low activities of daily life (ADL) at admission**	**53 (58.2)**	**5 (3.5)**	**14 (4.2)**	**<0.001**	**<0.001**	**0.907**
**Low ADL at discharge**	**25 (27.5)**	**2 (1.4)**	**4 (1.2)**	**<0.001**	**<0.001**	**1**
**Left ventricular (LV) ejection fraction (EF) (%)**	**47.2±16.1**	**37.7±18.0**	**51.3±18.3**	**<0.001**	**0.038**	**<0.001**
**HF with reduced EF**	**31 (34.1)**	**79 (55.6)**	**87 (26.4)**	**0.002**	**0.194**	**<0.001**
**HF with mid-range EF**	**23 (25.3)**	**30 (21.1)**	**61 (18.5)**	**0.564**	**0.203**	**0.600**
**HF with preserved EF**	**37 (40.7)**	**33 (23.2)**	**181 (55.0)**	**0.007**	**0.021**	**<0.001**
**LV end-diastolic volume (mL)**	**115.5±45.3**	**161.9±69.7**	**111.4±49.5**	**<0.001**	**0.374**	**<0.001**
**LV end-diastolic diameter (mm)**	**47.7±8.5**	**56.3±10.6**	**47.4±9.2**	**<0.001**	**0.751**	**<0.001**
**LV end-systolic diameter (mm)**	**36.4±9.5**	**46.2±13.2**	**35.0±11.0**	**<0.001**	**0.137**	**<0.001**
**Left atrial diameter (mm)**	**43.0±7.3**	**45.9±7.2**	**45.2±9.1**	**0.004**	**0.020**	**0.383**
**Mitral regurgitation**	**1.5±1.0**	**1.5±1.0**	**1.6±1.1**	**0.715**	**0.798**	**0.447**
**Tricuspid regurgitation**	**1.1±0.8**	**1.0±0.8**	**1.5±1.1**	**0.384**	**0.001**	**<0.001**
**Right ventricular systolic pressure (mmHg)**	**28.7±9.0**	**25.9±8.8**	**30.7±10.9**	**0.012**	**0.250**	**<0.001**
**Atrial septal defect after ablation**	**2 (2.2)**	**3 (2.1)**	**25 (7.6)**	**1**	**0.106**	**0.036**
**Aortic valve replacement**	**3 (3.3)**	**2 (1.4)**	**22 (6.7)**	**0.384**	**0.337**	**0.031**
**Hemodialysis**	**21 (23.1)**	**0 (0)**	**0 (0)**	**<0.001**	**<0.001**	**1**
**Angiotensin-converting enzyme inhibitor/Angiotensin receptor blocker**	**37 (40.7)**	**52 (36.6)**	**179 (54.4)**	**0.631**	**0.028**	**<0.001**
**Beta blockers**	**34 (37.4)**	**54 (38.0)**	**198 (60.2)**	**1**	**<0.001**	**<0.001**
**Mineralocorticoid receptor antagonist**	**8 (8.8)**	**27 (19.0)**	**114 (34.7)**	**0.052**	**<0.001**	**<0.001**
**Diuretic**	**33 (36.3)**	**55 (38.7)**	**214 (65.1)**	**0.810**	**<0.001**	**<0.001**
**Direct oral anticoagulants /Warfarin used at admission**	**19 (20.9)**	**39 (27.5)**	**150 (45.6)**	**0.328**	**<0.001**	**<0.001**
**Direct oral anticoagulants /Warfarin used at discharge**	**22 (24.2)**	**71 (50.0)**	**179 (54.4)**	**<0.001**	**<0.001**	**0.436**
**Estimated glomerular filtration rate (eGFR) at admission (mL/min/1.73m^2)**	**32.6±27.1**	**54.5±17.7**	**40.6±18.3**	**<0.001**	**0.009**	**<0.001**
**eGFR at discharge (mL/min/1.73m^2)**	**32.7±29.0**	**50.0±16.4**	**39.0±19.1**	**<0.001**	**<0.001**	**<0.001**

**Creatinine clearance rate (Ccr) at admission (mL/min)**	**19.8±13.1**	**69.7±30.8**	**33.3±15.5**	**<0.001**	**<0.001**	**<0.001**
**Ccr at discharge (mL/min)**	**20.2±13.3**	**63.0±30.3**	**31.9±14.5**	**<0.001**	**<0.001**	**<0.001**
**Creatinine at admission (mg/dL)**	**2.7±2.5**	**1.1±0.4**	**1.3±0.6**	**<0.001**	**<0.001**	**<0.001**
**Creatinine at discharge (mg/dL)**	**3.0±3.1**	**1.2±0.5**	**1.4±0.7**	**<0.001**	**<0.001**	**<0.001**
**Albumin (g/dL)**	**3.2±0.5**	**3.6±0.4**	**3.4±0.5**	**<0.001**	**0.001**	**<0.001**
**Total protein (g/dL)**	**7.0±0.7**	**7.0±0.6**	**7.0±0.6**	**0.993**	**0.711**	**0.653**
**C-reactive protein (mg/dL)**	**4.1±5.5**	**1.4±2.5**	**2.2±4.0**	**<0.001**	**<0.001**	**0.412**
**Hemoglobin (g/dL)**	**11.0±2.1**	**13.6±2.5**	**11.4±2.0**	**<0.001**	**0.164**	**<0.001**
**Systolic blood pressure (SBP) at admission (mmHg)**	**137.5±27.1**	**131.4±25.9**	**130.8±25.4**	**0.091**	**0.038**	**0.831**
**SBP at discharge (mmHg)**	**110.0±20.4**	**109.8±16.2**	**113.9±18.0**	**0.936**	**0.103**	**0.016**
**Diastolic blood pressure (DBP) at admission (mmHg)**	**79.7±69.0**	**76.6±20.1**	**69.3±16.3**	**0.280**	**0.08**	**<0.001**
**DBP at discharge (mmHg)**	**56.0±11.1**	**62.0±9.3**	**59.1±10.1**	**<0.001**	**0.017**	**0.003**
**Heart rate at admission (bmp)**	**86.9±21.0**	**94.6±26.9**	**80.8±20.6**	**0.016**	**0.014**	**<0.001**
**Heart rate at discharge (bmp)**	**69.7±16.8**	**69.8±13.1**	**70.5±13.3**	**0.966**	**0.687**	**0.605**

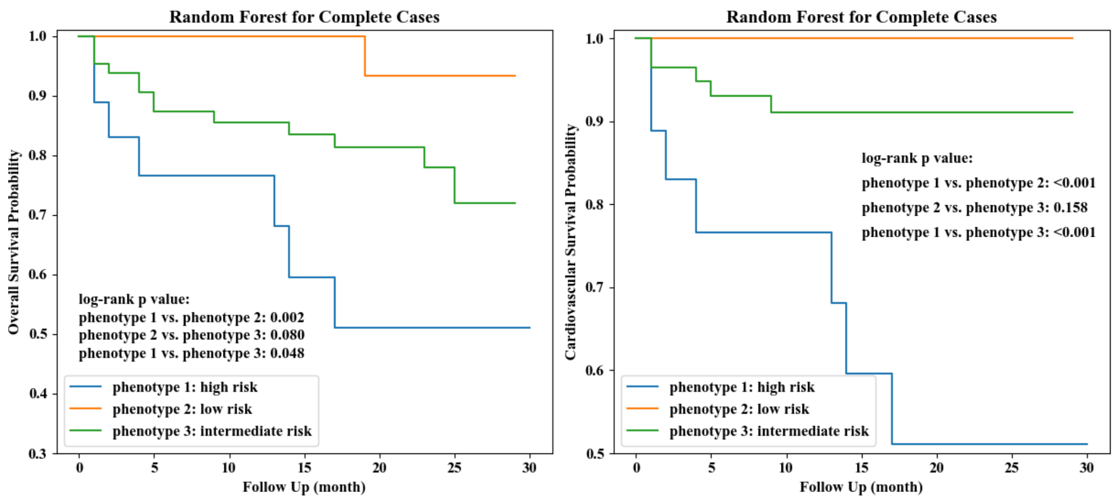

Figure S2. Survival curves of phenotypes classified by random forest for complete cases in validation dataset.
